# Delayed Hypothalamic Dysfunction Presenting as Recurrent Hypothermia After Treatment of Suprasellar Germinoma: A Case Report

**DOI:** 10.1002/ccr3.73535

**Published:** 2026-09-24

**Authors:** Ayumi Nishimura, Tomonobu Kado, Shiho Fujisaka, Masaru Kato, Manabu Ishiki

**Affiliations:** ^1^ Faculty of Education and Research Promotion, Health Administration Center University of Toyama Toyama Japan; ^2^ First Department of Internal Medicine, Graduate School of Medicine and Pharmaceutical Science University of Toyama Toyama Japan

**Keywords:** germinomas, hypothalamic diseases, hypothermia, radiotherapy

## Abstract

Long‐term survivors of suprasellar germinomas may develop delayed hypothalamic dysfunction, presenting as recurrent hypothermia after years of apparently normal thermoregulation. Recognition of this delayed complication and proactive thermal management are essential to prevent severe hypothermic episodes.

## 1 Introduction

Intracranial tumors arising in the suprasellar region or involving the hypothalamus are among the major causes of hypothalamic dysfunction [1]. Such dysfunction may also occur as a late complication of cranial radiotherapy [2, 3, 4, 5, 6].

The hypothalamus is critical for thermoregulatory homeostasis, and its dysfunction can lead to central hyperthermia or hypothermia [1, 7]. Herein, we report a case of hypothalamic dysfunction presenting as recurrent hypothermia during long‐term follow‐up after chemoradiotherapy for a suprasellar germinoma.

## 2 Case History/Examination

**FIGURE 1:**
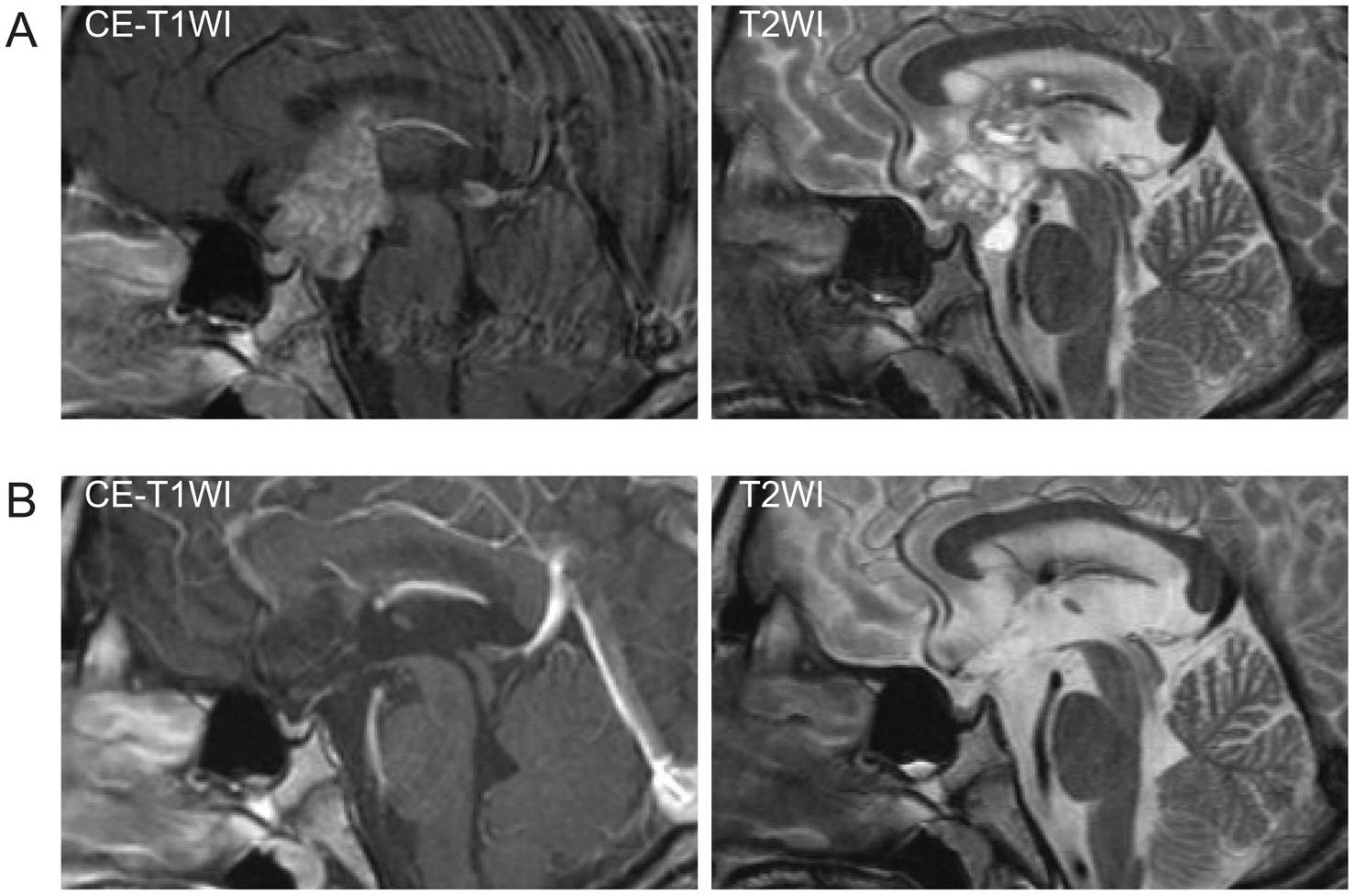
Brain magnetic resonance imaging (MRI) before and after treatment. (A) Pre‐treatment contrast‐enhanced T1‐ and T2‐weighted MRI demonstrating a suprasellar mass extending toward the third ventricular and pineal regions, involving the hypothalamic region. (B) Post‐treatment contrast‐enhanced T1‐ and T2‐weighted MRI after completion of chemoradiotherapy showing marked tumor regression.

A 24‐year‐old Japanese woman had initially presented at 13 years of age to a local clinic with fatigue and fever. Laboratory evaluation revealed hypothyroidism and hypernatremia, raising suspicion of a pituitary lesion. Cranial computed tomography (CT) demonstrated a suprasellar mass, and she was referred to the pediatric department. A biopsy was not performed because of the high risk of injury to the surrounding brain structures. However, given a mildly elevated serum β‐human chorionic gonadotropin (β‐hCG) level (0.3 ng/mL) and magnetic resonance imaging (MRI) findings (Figure 1A), she was clinically diagnosed with primary intracranial germinoma. At diagnosis, the patient had panhypopituitarism and central diabetes insipidus.

The patient received chemotherapy (CARE regimen) followed by radiotherapy (whole‐ventricular irradiation, 23.4 Gy in 13 fractions). Follow‐up brain MRI after completion of chemoradiotherapy demonstrated marked tumor regression (Figure 1B), and serum β‐hCG levels normalized (≤ 0.1 ng/mL).

Before treatment, she had persistent hyperthermia with body temperatures ranging from 37°C to 38°C. No clinical or laboratory evidence of infection or inflammation was identified, and thermoregulatory dysfunction secondary to hypothalamic involvement was suspected. Approximately 1 month after completion of therapy, her body temperature stabilized at 36°C–37°C. Thereafter, transient mild temperature elevations occasionally occurred during summer but did not interfere with daily activities.

At 17 years of age, she transitioned to adult endocrine care and continued hormone replacement therapy with hydrocortisone, levothyroxine, growth hormone, and desmopressin. Secondary hypogonadism was managed with sex hormone replacement therapy in the gynecology department.

At 21 years of age (February), she presented with fatigue, abdominal pain, and poor oral intake. Laboratory tests revealed hyponatraemia (131 mEq/L), hypoglycaemia (66 mg/dL), and relative adrenal insufficiency due to a sick‐day state; therefore, stress‐dose steroids were administered. However, the serum cortisol level measured at presentation, which later became available, was elevated at 56.2 μg/dL. Her body temperature at presentation was 34.5°C, and she was admitted for active external warming. Her symptoms improved, and she was discharged on Day 7 of hospitalization.

At 22 years of age (January), she again presented with fatigue and was found to have hypothermia (33.3°C) and bradycardia (heart rate, 41 beats/min). In March of the same year, she was readmitted because of fatigue and decreased responsiveness; her body temperature was 35.2°C. Stress‐dose steroid supplementation was administered; however, no rapid symptomatic improvement was observed. The serum cortisol level on admission, which was later confirmed, was elevated at 42.1 μg/dL. Brain MRI revealed no tumor recurrence or new lesions. Hypothermia in the 34°C range persisted during hospitalization, but her general condition gradually improved with active external warming, and she was discharged on hospital Day 11.

## 3 Differential Diagnosis, Investigations and Treatment

Although adrenal insufficiency was initially suspected during both hypothermic episodes, elevated serum cortisol levels and the lack of response to steroid supplementation made acute adrenal crisis unlikely. Based on these clinical findings and the absence of tumor recurrence on brain MRI, delayed hypothalamic dysfunction was considered the most likely cause of her recurrent hypothermia.

## 4 Outcome and Follow‐Up

Given this clinical course, hypothermia was considered the primary cause of her clinical deterioration and impaired consciousness. Therefore, during the winters at 23 and 24 years of age, proactive thermal management in daily life was recommended, including maintaining room temperature above 20°C, using an electric blanket, and consuming warm beverages. Since the implementation of these measures, she has had no episodes of clinical deterioration or decreased consciousness requiring hospitalization.

## 5 Discussion

This case illustrates hypothalamic dysfunction characterized by recurrent hypothermia manifesting approximately 8 years after chemoradiotherapy for suprasellar germinoma. Although thermoregulatory disturbances are recognized manifestations of hypothalamic injury, reports describing hypothermia as the predominant and recurrent clinical feature after a prolonged remission remain scarce.

In this patient, the tumor extensively involved the suprasellar region, third ventricle, and pineal region, suggesting direct hypothalamic infiltration at diagnosis. Consistent with this anatomical involvement, she exhibited persistent hyperthermia of approximately 38°C despite the winter season, indicating hypothalamic thermoregulatory dysfunction. Although the hyperthermia resolved following chemoradiotherapy and she remained clinically stable for several years, she subsequently developed severe hypothermia that required hospitalization approximately 8 years later. This delayed transition from the initial hyperthermia to recurrent profound hypothermia was the defining feature of this case.

Hypothalamic dysfunction can develop as a late complication of cranial radiotherapy, with clinical manifestations emerging months to decades after treatment in a dose‐ and time‐dependent manner [2, 3, 4, 5, 6]. Furthermore, the hypothalamus is more radiosensitive than the pituitary gland and may be functionally impaired even at relatively low doses [3, 5]. In the present case, whole‐ventricular irradiation at a total dose of 23.4 Gy may have contributed to delayed hypothalamic dysfunction presenting as recurrent hypothermia.

Although hypothermia can exacerbate adrenal insufficiency and warrants careful attention, serum cortisol levels during hypothermic episodes were elevated in this patient, arguing against overt adrenal insufficiency [1]. The hyponatraemia and hypoglycaemia observed during these episodes were therefore more likely attributable to factors associated with hypothermia itself, including reduced oral intake, impaired hepatic gluconeogenesis, and attenuation of counterregulatory hormonal responses, such as glucagon and sympathetic activation [1, 8, 9, 10].

For hypothermia associated with hypothalamic dysfunction, several pharmacological agents targeting neurotransmitter systems have been reported to be beneficial in select cases; however, no established therapy exists, and supporting evidence remains limited [1, 7, 11]. Accordingly, active external warming constitutes the mainstay of management. This includes direct warming measures, such as electric blankets and hot water bottles, as well as environmental temperature control using heating devices [1].

In the present case, proactive thermal management during winter prevented further episodes of severe deterioration and impaired consciousness. In long‐term survivors of brain tumors, follow‐up should address not only endocrine function but also thermoregulatory capacity, as hypothalamic thermoregulatory dysfunction may emerge years after apparently successful treatment. Practical lifestyle guidance plays an important role in preventing severe complications of delayed hypothalamic dysfunction.

## Author Contributions

**Ayumi Nishimura:** writing – original draft, conceptualization, investigation. **Tomonobu Kado:** investigation, writing – review and editing. **Shiho Fujisaka:** investigation, writing – review and editing. **Masaru Kato:** investigation, writing – review and editing. **Manabu Ishiki:** investigation, writing – review and editing.

## Funding

The authors have nothing to report.

## Consent

Written informed consent was obtained from the patient for publication of this case report.

## Conflicts of Interest

The authors declare no conflicts of interest.

## Data Availability

Data sharing is not applicable to this article.
